# Repair of Physiologic Time Series: Replacement of Anomalous Data Points to Preserve Fractal Exponents

**DOI:** 10.3389/fbioe.2017.00010

**Published:** 2017-02-21

**Authors:** Mark Shelhamer, Steven B. Lowen

**Affiliations:** ^1^Department of Otolaryngology – Head and Neck Surgery, The Johns Hopkins University School of Medicine, Baltimore, MD, USA; ^2^Department of Biomedical Engineering, The Johns Hopkins University School of Medicine, Baltimore, MD, USA; ^3^Department of Psychiatry, McLean Hospital, Harvard Medical School, Belmont, MA, USA

**Keywords:** fractal physiology, power-law scaling, data outliers, spectral slope, simulated data

## Abstract

Extraction of fractal exponents *via* the slope of the power spectrum is common in the analysis of many physiological time series. The fractal structure thus characterized is a manifestation of long-term correlations, for which the temporal order of the sample values is crucial. However, missing data points due to artifacts and dropouts are common in such data sets, which can seriously disrupt the computation of fractal parameters. We evaluated a number of methods for replacing missing data in time series to enable reliable extraction of the fractal exponent and make recommendations as to the preferred replacement method depending on the proportion of missing values and any *a priori* estimate of the fractal exponent.

## Introduction

Since the brilliant and defining work of Mandelbrot on fractal mathematics (Mandelbrot, 1999) and the discovery of fractal structure in firing rates of auditory neurons (Teich, 1989), fractal physiology has become an area of great research interest (Bassingthwaighte et al., 1994). By *fractal structure* we mean, most generally, self-similarity: the repeating of patterns at different spatial or temporal scales. (In reference to time series, this is more properly termed *self-affinity*; see below.) In terms of time-series analysis, fractal structure is most often manifested statistically: variability or fluctuations change in a systematic manner with the duration of a temporal window into the data record. This was first found by Hurst in a study of the series of annual peak Nile River levels and quantified in terms of a new parameter known as the rescaled range, which is the range of the data divided by the SD (R/S). This was found to be proportional to the duration of the data record (T, in years), raised to a power: R/S ~ T*^H^* (Hurst, 1951). This reflects *power-law* behavior, where the variability is a power-law function of time scale; for values of *H* other than 0.5, it also means that the system has memory, such that future values are dependent on previous ones.

Strictly speaking, the concept of self-similarity does not apply to functions, including those represented by time series as studied here. Instead, *self-affine* is the correct term for fractal characteristics in a time series, which refers to scaling that can be different along the abscissa and the ordinate (behaviors along the two axes are independent). Self-similarity can only apply where both axes are subject to the same metric.

In more general terms, power-law scaling is often reflected in the autocorrelation function of the time series, which decays as a power-law function of time lag: R(τ) ~ 1/τ^β^. Under broad conditions, the associated power spectrum (the Fourier transform of the autocorrelation function) also exhibits power-law decay: S(f) ~ 1/f^α^ (Rangarajan and Ding, 2000), which leads to a clear interpretation: as temporal scale (inverse frequency) increases (frequency decreases) by a factor of *x*, the fluctuations at that scale increase by a factor of *x*^α^, over a broad range of temporal (frequency) scales. While the physical or physiological meaning of this scaling is not completely understood, the finding is ubiquitous in biological and physiological systems (Gisiger, 2001). There are many ways to characterize this fractal structure; one of the most straightforward in terms of computation and interpretation is the scaling exponent of the power spectrum (α), easily obtained from a linear regression on a log–log plot of the periodogram, which is an estimate of the power spectrum S(f) (Lowen and Teich, 2005).

It is important to note that analysis of a time series for such fractal (power-law) scaling is based on temporal structure: the temporal ordering of the time points is critical. This can be problematic when dealing with physiological time series which often have artifacts that lead to missing data points (ectopic beats in EKG, blinks in eye-movement records, etc.). We wanted to determine if there are ways to replace missing data points—for which their times of occurrence have been corrupted—that do not significantly alter the computed value of the fractal exponent. We assume that bad (missing) data points can be identified as such through other means, such as altered waveform morphology, but that their timing is not so easily identifiable. Thus, when reconstructing the time series and inserting the missing data, it is the *timing* of that data that is crucial. We specifically focus on *fractal point processes*, where the timing of nearly identical, stereotyped, events is of interest (eye blinks, QRS complexes, gait intervals, etc.). Thus, we are concerned with replacing missing data points by inserting new values in the correct place temporally with respect to the rest of the time series.

A number of approaches to this problem have been proposed. Most are in the area of heart-rate variability (HRV), where timing of R waves is critical to a variety of linear and non-linear measures. Lippman et al. (1994) compared four methods to correct for ectopic beats that had been artificially inserted into human heartbeat data and examined their effects on measures of HRV. (Ectopic beats are extra or skipped beats that arise from other than the normal cardiac electrical conduction mechanism. These variations in the normal rhythm are generally benign and can be ignored if the “typical” cardiac timing mechanism is of interest.) The methods they examined were simple deletion, linear interpolation, cubic spline interpolation, and pattern matching. They found that “deletion and non-linear predictive interpolation performed superiorly to linear or cubic spline interpolation, which overestimated low-frequency power and underestimated high-frequency power.” Another group evaluated methods for first detecting which beats are ectopic and then ameliorating their effects by replacing them using a model of heart timing, which is an established model that relies on physiological limits in HR bandwidth to provide bounds on the possible change in rate from one beat to the next (Mateo and Laguna, 2003). In another study of replacement methods for missing (ectopic) beats, interpolation yielded better performance than did deletion, which in turn was better than no editing of ectopic beats, for a variety of non-linear measures except entropy (Tarkiainen et al., 2007). Methods for determining which beats are ectopic and then accurately restoring data integrity have been evaluated in terms of both accuracy and computational cost (Colak, 2008). Note that while ectopic cardiac beats can be determined due to the particular characteristics of the human heartbeat, our approach is more general.

In the case of neural spike trains, explicitly accounting for spike jitter can yield more robust characterizations of spike-train patterns. In one approach (Aldworth et al., 2005), an iterative process was used to adjust timing jitter in recorded spike trains, in which each candidate de-jittered signal was compared *via* a distance measure to the mean of the de-jittered signals on the previous iteration, until convergence was obtained. A model-based approach was used to make the putative timing adjustments, which made reasonable assumptions about the possible jitter that occurs in a sequence after a stimulus. This is different from the approach that we pursue, where we desire to replace isolated samples with unknown timing, over an entire time series.

We examined a number of methods for dealing with missing data values, specifically in the context of how various replacement algorithms affect the resulting estimates of the fractal exponent. Recommendations for which method to use, based on the proportion of data values to be replaced and the likely range of the fractal exponent *a priori*, are provided.

## Methods

The algorithms described below are provided in Supplementary Material. The computer code includes routines for the generation of the simulated data. The retina, geniculate, auditory nerve, and saccade data sets are available on request from the authors. The HRV data are available as data set 16273 on the Physionet archive (http://physionet.org).

### Overview

We examined the performance (in terms of the effect on computed fractal exponent) of several different data-replacement algorithms on simulated data with known exponents and on actual physiological data. Our approach for simulated data was to generate sets of data values (event times) with known power-law characteristics, delete some of the values (while leaving adjacent values intact), and then replace those missing values using a variety of algorithms. We evaluated performance by estimating the power-law exponent of the power spectral density (“fractal exponent”) before and after deletion and replacement and comparing those exponents. We repeated this approach with real physiological data. (Note that some of our algorithms are similar to those used in previous studies. For example, in the Lippman investigation described above, they used deletion which is similar to our method RR, linear interpolation which is similar to our HH, and pattern matching which is similar to our NX and SX.)

### Simulation Parameters

We varied two parameters in the generation of our simulated data sets. First, the intended power-law exponent, α, was 0, 0.5, 1.0, 1.5, or 2.0. Second, the probability distribution of the values (event times) was either exponential, Gaussian, Laplace, mixed, or uniform. The mixed distribution was randomly selected: Gaussian with probability 3/4, and de-meaned exponential with probability 1/4 (both zero-mean and unit-variance). These distributions were chosen to span the ranges of higher moments seen in the real data that were analyzed. The proportions of events deleted and replaced, *p*, were 0, 1, 2, 5, 10, or 20%. Finally, 10 repair algorithms were used (see below). This resulted in a total of 1,500 unique combinations of data type and repair method.

There are two ways to categorize the data in which we are interested. First, the data can be real or simulated. Second, the data can be interval-based (the values represent time between events) or event-based (the values represent the times of the events). We study all four possible combinations of data types in this paper. For interval-based data, when an interval is deleted, there is no information in the remaining data as to what the numerical value of that interval was; the record of that interval is simply removed. This applies to lists of intervals, such as heartbeat data or neural spike-train recordings, where there is no external reference stimulus associated with the events demarking the intervals (and hence stimulus–response latency has no meaning). In contrast, in event-based recordings, events are deleted rather than intervals. Deleting a single event eliminates two intervals, but retains their sum, so that some local information remains regarding the pre-deletion values of those intervals. This applies to lists of events such as eye movements in response to visual targets. Here, there is an external stimulus event associated with each recorded event, and typically the time between stimulus and response (the latency) is recorded rather than the times between successive responses. (We note that the interval/event distinction is largely one of data storage, and so heartbeat data stored as beat times would fall into the event-based type.) Deletion and repair methods necessarily differ for interval- and event-based data types.

### Algorithms Tested

For event-based data, we tested the following repair and replacement algorithms:

- HH: replace the missing event with a value that represents the time midway between the previous and following events. This posits no change in event timing for the three consecutive events.
- RR: remove the missing event, yielding a time series two points shorter for each missing event. Each event forms the terminus for two intervals, so removing an event without replacing it necessitates removing the two intervals flanking it. The two dangling events thus created are then merged: duration of the double interval flanking the missing event is computed, and that value is subtracted from all events after the missing event. The new time of the first event after the missing event then coincides with the (untouched) value of the last event before the missing event.
- FF: throughout the entire data set, identify all legitimate intervals (those which have valid, non-deleted, events defining them). For each adjacent pair of such intervals, find the ratio of the earlier interval to the later interval, and select one such ratio at random. Insert a new event (data value) in place of the missing one, such that the ratio of the two new intervals thus created matches the target ratio. This attempts to reproduce any local temporal trend of increasing or decreasing rate.
- NX: throughout the entire data set, identify all legitimate intervals, as above. For each consecutive sequence of *X* such intervals in the data set, find that sequence that best matches the neighborhood of the missing event (produces the lowest mean-squared error when compared to the sequence of intervals surrounding the missing event), and insert the data from the best-fitting sequence in place of the missing value. The number of intervals on each side, *X*, is {0, 1, 2, or 3}. This algorithm assumes that any local trend might be repeated and so can serve as a template to replace missing values. (In more detail: take the *X* times between events on either side of the missing event, and match those 2*X* + 1 intervals to all possible sets of intervals in the rest of the data. To compare correctly, remove the central event in the candidate set of intervals to be matched. For the one that fits the best, use that central interval to restore the missing one in the target. As it won’t fit perfectly, scale the sum of the intervals around the match to that of the target, and then use those scaled intervals to place the missing event.)
- SX: similar to NX, but allow the entire template (sequence of *X* intervals) to be scaled by a multiplicative factor to obtain the best fit. Here, *X* is {1, 2, or 3}, as 0 permits all intervals to be scaled perfectly and thus is degenerate.

For interval-based data, we tested the following algorithms:

- HH: replace the missing interval with the average of all remaining intervals. This assumes that the best model of the data is essentially a noisy clock with a fixed rate.
- RR: remove the interval, yielding a time series one interval shorter for each missing interval. This simply allows the missing interval data to remain missing, and so invariably disrupts the chronology of the data points.
- FF: replace the missing interval with one randomly selected from those remaining. This is similar to HH but has a preference for values that are prevalent in the data set.
- NX: throughout the data set, identify all legitimate (non-deleted) intervals. For all intervals which have at least *X* legitimate intervals on either side (that is, for all sequences of 2*X* + 1 intervals), find the sequence that best matches the neighborhood of the missing interval, and insert that best-fitting interval in place of the missing one. The number of intervals on each side *X* is {0, 1, 2, or 3}. This again attempts to identify and reproduce local trends in timing.
- SX: similar to the above, but allow the entire template to be scaled for the best fit. Here, *X* is {1, 2, or 3}, as 0 permits all intervals to be scaled perfectly and thus is degenerate.

### Procedure: Simulated Data

Simulated data sets were generated and analyzed with the following procedure, where “value” stands for either an event or an interval, depending on the data being generated.

For the generation of each simulated data set (time series), there is an intended value of fractal exponent α, chosen from the set described previously (0, 0.5, 1.0, 1.5, 2.0). The particular choice of probability distribution will affect the generated fractal exponent slightly, as may retaining only part of a longer simulation in order to reduce periodicity effects. Thus, a revised target value, α_0_, was created for each intended value of α and each probability distribution, as follows:

(1) Generate an array of 65,536 values, with a target 1/f^α^ periodogram, using the random-phase method (Theiler et al., 1992).
(2) Retain only the first 1,024 values to reduce periodicity effects.
(3) Generate 1,024 random values with the desired probability distribution.
(4) Sort the values from step 3 so that they have the same relative ordering as those of step 2, and discard the values from step 2. The result is a sequence of data values (events or inter-event intervals) with the desired distribution and which has an approximately 1/f^α^ periodogram.
(5) Calculate the periodogram of the new data set using a Hann window, and estimate the power-law exponent, α_1_, using a least-squares fit on a doubly logarithmic plot (Lowen and Teich, 2005).
(6) Repeat this process 1,000 times, with different random seeds.
(7) Iteratively adjust α_0_ until the average of α_1_ over all 1,000 runs is as close to α as possible.

With this value of α_0_ in hand (for a desired ultimate value of α), simulated data sets were generated and analyzed as follows (for each set of parameters α and *p*, each distribution, and each replacement algorithm):

(1) Generate an array of 65,536 values, with a $1/{\text{f}}^{\alpha_{0}}$ periodogram, using the random-phase method.
(2) Retain only the first 1,024 values to reduce periodicity effects.
(3) Generate 1,024 random values with the desired probability distribution.
(4) Sort the values from step 3 so that they have the same relative ordering as those of step 2, and discard the values from step 2. The result is a sequence of data values (events or inter-event intervals) with the desired distribution and which has an approximately 1/f^α^ periodogram.
(5) Calculate the periodogram of the data set using a Hann window, and estimate the pre-deletion power-law exponent, α_1_, using a least-squares fit on a doubly logarithmic plot (Lowen and Teich, 2005).
(6) Delete a proportion *p* of randomly selected values from the data set, where *p* is the proportion chosen for the particular trial. Two or more values in a row are never deleted.
(7) Replace the missing values using the desired replacement algorithm.
(8) Recalculate the periodogram and estimate the post-repair power-law exponent, α_2_.
(9) Differences between α_1_ (pre-deletion) and α_2_ (after deletion and replacement) are computed, quantifying the effects of the deletion and replacement process.
(10) Repeat this process 1,000 times with different random seeds.
(11) For all values of *p*, the last periodogram calculated (from either step 5 or 8) is averaged over all 1,000 runs for display purposes.

Thus, there are four values of alpha: (a) without a subscript for the design value, (b) a subscript of 0 for the value used in the simulations, (c) a subscript of 1 for the value measured from the simulations (of which there are 1,000), and (d) a subscript of 2 for the result after deletion and repair (there are 1,000 of these as well).

The first four steps above form a method related to the amplitude-adjusted Fourier transform algorithm (AAFT) for the generation of surrogate data sets (Theiler et al., 1992). Such a one-pass method can yield spurious spectra (Schreiber and Schmitz, 1996). However, our resulting spectra so closely approximated the design values that further iterations were not necessary (see Results). Note also that the power-law exponent of the average of the periodograms differs from the average of the power-law exponents of the periodograms due to the non-linearity involved in calculating the power-law exponent.

### Procedure: Real Data

The same replacement algorithms were also applied to several sets of real physiological data. See Table 1 for the interval data sets and Table 2 for the event data sets. Data sets on intervals between neural firings in retinal ganglion cells and in lateral geniculate nucleus cells were recorded with single-unit electrodes (Teich et al., 1997; Lowen et al., 2001), as were recordings from auditory-nerve firing (Lowen and Teich, 1996). Normal and abnormal heartbeat (R-R interval) data are from an online archive (Goldberger et al., 2000). Real interval-based data generally exhibit power-law behavior only for time scales several times longer than the average inter-event time (Lowen and Teich, 2005). The number of intervals was divided by 1,024, and rounded down to the nearest integer, yielding a block size *b* (*b* ≥ 23 in all cases). Adjacent, non-overlapping blocks of *b* intervals were summed, yielding 1,024 aggregated values. Remaining intervals were removed from further analysis.

**Table 1:** **Properties of interval-based data sets used here**.

Properties of original data		Properties of aggregate			
Data type	Intervals	Sum	Skewness	Excess kurtosis	Exponent
Retinal ganglion firing	120,714	117	0.1	−1.6	0.860
Thalamus (LGN) firing	24,285	23	2.1	6.4	0.781
Auditory nerve fiber firing	127,505	124	1.0	1.7	0.773
	90,804	88	0.9	0.7	0.791
Heartbeat (abnormal)	80,878	78	1.7	3.5	1.739
Heartbeat (normal)	88,140	86	0.2	−1.0	1.508

*Data were aggregated into 1,024 non-overlapping sums, each of which consisted of the number of original intervals given in column 3. Higher moments of the aggregated data (skewness and excess kurtosis) were computed to guide the selection of distributions for simulated data*.

**Table 2:** **Properties of event-based data sets used here**.

Driving period (s)	Events	Skewness	Excess kurtosis	Exponent
0.56	998	0.5	2.6	0.891
0.56	999	0.2	−0.4	0.942
0.56	996	0.1	1.0	0.895
1.67	1,000	0.0	3.7	0.242
1.67	998	2.1	5.1	0.198
1.67	996	0.6	1.2	0.338

*All are human saccadic eye-movement recordings. Driving periods <0.6 s yield predictive saccades, while those >1.6 s yield reactive saccades. Data were not aggregated. Higher moments of the data (skewness and excess kurtosis) were computed to guide the selection of distributions for simulated data*.

The real event-based (as opposed to interval-based) data are from experiments on saccadic eye movements (Shelhamer and Joiner, 2003) For these data, human subjects were presented with two visual targets that alternated at a low rate (target jump every 1.67 s, a rate of 0.3 Hz), and a high rate (target jump every 0.56 s, a rate of 0.9 Hz). The low rate generates reactive saccadic eye movements that take the eyes rapidly from one target to the other, with high latencies. The series of consecutive latencies forms a time series with a low fractal exponent since the values are not strongly correlated. At the high pacing rate, saccades are predictive and consecutive latencies are strongly correlated, with higher exponents.

For real data, steps 1–4 in the analysis above do not make sense and so are not performed. Furthermore, as there is only one realization available, only the deletion process varies across runs; for simulated data, the pre-deletion values vary across runs as well. Otherwise, the procedure follows that for simulated data.

**Figure 1:**
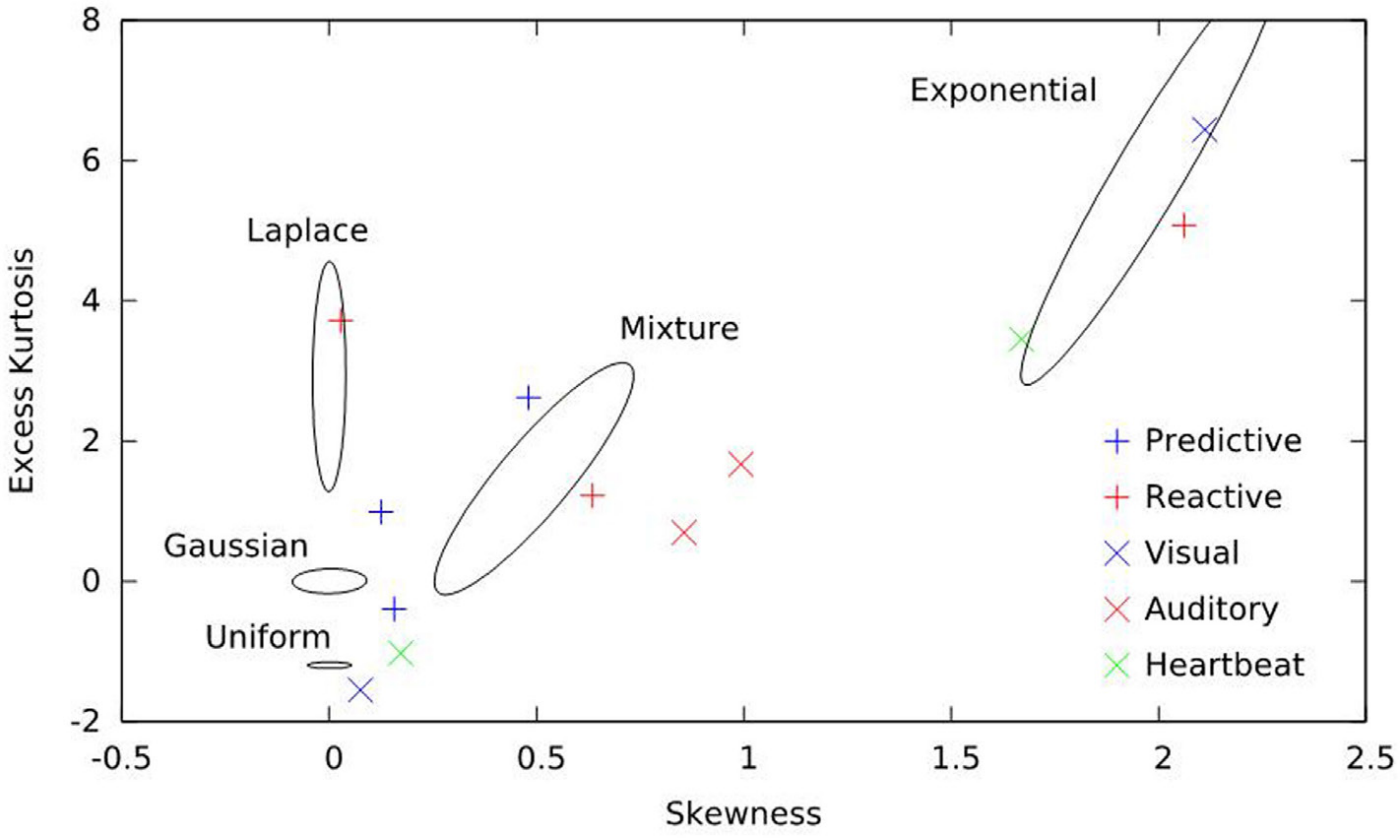
**Skewness and excess kurtosis of the real data sets and simulations employed here**. Real data are represented by symbols and consist of predictive saccades, reactive saccades, auditory nerve-fiber firings, visual-system neuron firings, and heartbeats. Simulated data are represented by curves; for each distribution, the smallest ellipse that encompasses 50% of the simulated results is shown. Together, the five distributions span the higher-order moments of the real data sets.

The periodogram, an estimate of the power spectrum, is a second order quantity and, therefore, does not directly depend on higher moments of the data. Power-law exponents calculated from the periodogram inherit this property. However, deletion and repair is a non-linear process, and so differing higher moments in the original data could yield different measured exponents of the repaired data. We, therefore, chose distributions for simulation that span the ranges of skewness and kurtosis seen in the real data analyzed. For each distribution simulated, we computed the sample skewness and kurtosis for 1,000 realizations and generated contour plots; all were elliptical. We, then, constructed the smallest ellipse that enclosed 50% of those values and plotted those ellipses along with the skewness and kurtosis of the real data sets analyzed in this paper. Figure 1 presents the result and indicates that these parameters of the real data are well represented by the simulated distributions.

## Results

### General

Pre-deletion power-law exponents α_1_ were very close to the design values α; out of 25 α-distribution pairs, the worst-case error was 48 × 10^−6^ and all but four were within 2 × 10^−6^. Furthermore, periodograms averaged over all 1,000 runs very closely followed pure power-law forms. Correlation coefficients between log frequency and log periodogram values averaged over all 1,000 runs for the 20 α-and-distribution pairs with α ≠ 0 ranged from 0.996562 to 0.999800, averaging 0.998860, which thus validates our use of the single-pass AAFT-like algorithm.

**Figure 2:**
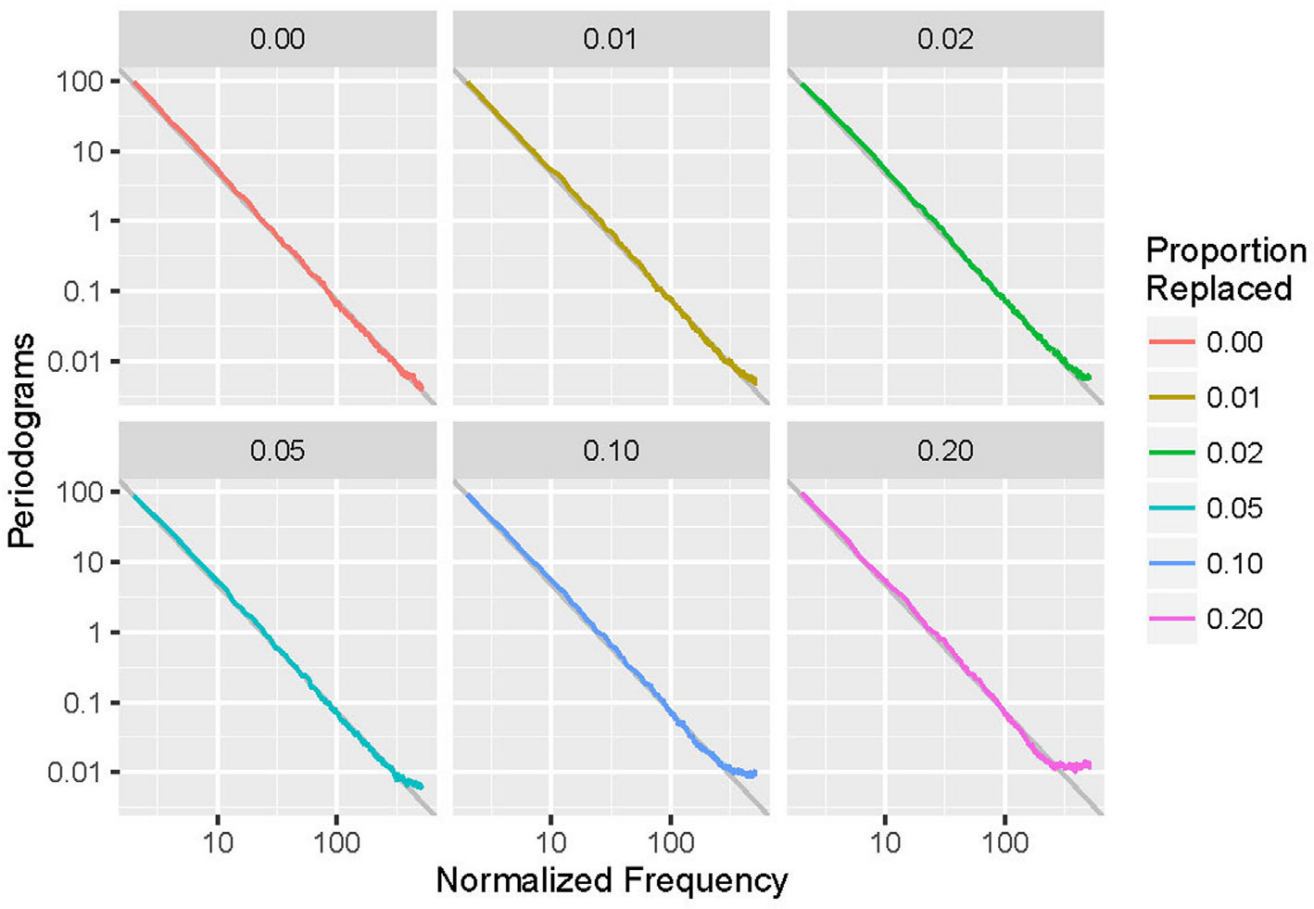
**Averaged periodograms of simulated event-based recordings with α = 2.0 and exponential distribution, with a variety of proportions (*p*) of events deleted and repaired using the N0 method**. Note that increasing *p* leads to increased high-frequency power and departure from the 1/f^α^ power-law form.

Periodograms (averaged over all 1,000 runs) for simulated event-based data with parameters α = 2, N0 method, and an exponential distribution are shown in Figure 2 as an example in doubly logarithmic format. Different lines correspond to different values of *p*, the proportion of anomalous data points replaced. Each fit is the linear regression of the undeleted (*p* = 0) plot and closely matches the data. Note that increasing deletion leads to a spurious increase of energy at high frequencies.

**Figure 3:**
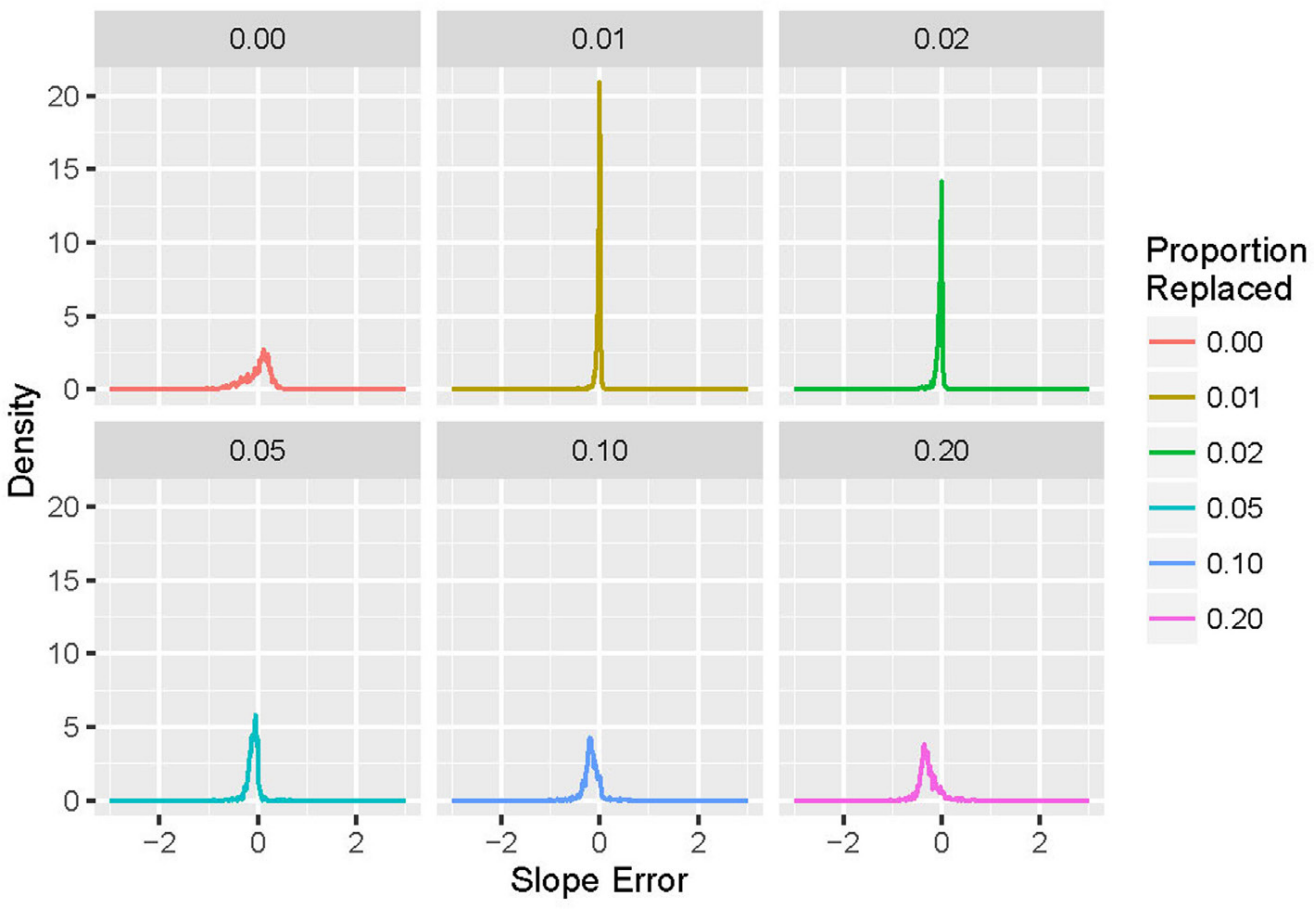
**Histograms of exponent error for simulated event-based recordings with α = 2.0 and an exponential distribution, with a variety of proportions (*p*) of events deleted and repaired using the N0 method**. For *p* = 0, the exponent is compared with the design value α = 2.0; for *p* > 0, the exponent is compared with the pre-deletion value. Note that increasing the proportion *p* leads to increases in both bias and variance in the measured post-repair power-law exponent.

For each run (of simulation and replacement), the power-law exponent was stored, and the results used to generate histograms of the difference between desired and actual generated values of exponent α. For *p* = 0 (no replacements), the difference from the target value of the power-law exponent (α_1_ − α) is plotted; for *p* > 0, the difference in the power-law exponent induced by deletion and replacement (α_2_ − α_1_) is displayed. An example is shown in Figure 3. Note that larger values of *p* lead to increases in both the bias and variance of α_2_.

For each set of parameters with *p* > 0, the root mean square error (RMSE) between α_1_ and α_2_ is computed as a summary statistic. A sample table for α = 2 and an exponential distribution is presented (Table 3). Each cell presents the RMSE for that parameter set, coded from white (zero) to red (worst). Note the steady decrease in performance as *p* increases (as more bad points are replaced), and consistent variation among the repair methods. (Here and throughout the text, “worst” and “worst-case” refer to the situation in which the RMSE between the actual exponent, α_1_, and the exponent after replacement by a particular algorithm, α_2_, is the largest, for a given parameter set or algorithm).

**Table 3:** **Root mean square error (RMSE) of the power-law exponent for the 10 repair methods vs the proportion of events deleted, *p*, for simulated event-based data with α = 2.0 and an exponential distribution**.

α = 2.0, Distribution is exponential						
R.M.	1%	2%	5%	10%	20%	Avg
NO	0.0672	0.0905	0.1440	0.2276	0.3316	0.1722
N1	0.0358	0.0534	0.0836	0.1249	0.2069	0.1009
S1	0.0352	0.0482	0.0912	0.1465	0.2040	0.1050
N2	0.0382	0.0535	0.0854	0.1315	0.2192	0.1056
S2	0.0322	0.0461	0.0735	0.1245	0.1818	0.0916
N3	0.0375	0.0556	0.0888	0.1419	0.2323	0.1112
S3	0.0342	0.0437	0.0751	0.1153	0.1771	0.0891
FF	0.0973	0.1847	0.2659	0.3919	0.5402	0.2960
HH	0.0377	0.0488	0.0748	0.1123	0.1585	0.0864
RR	0.0578	0.0793	0.1103	0.1575	0.2244	0.1259

*RMSE values are color coded, with white representing zero error and red the worst case*.

**Figure 4:**
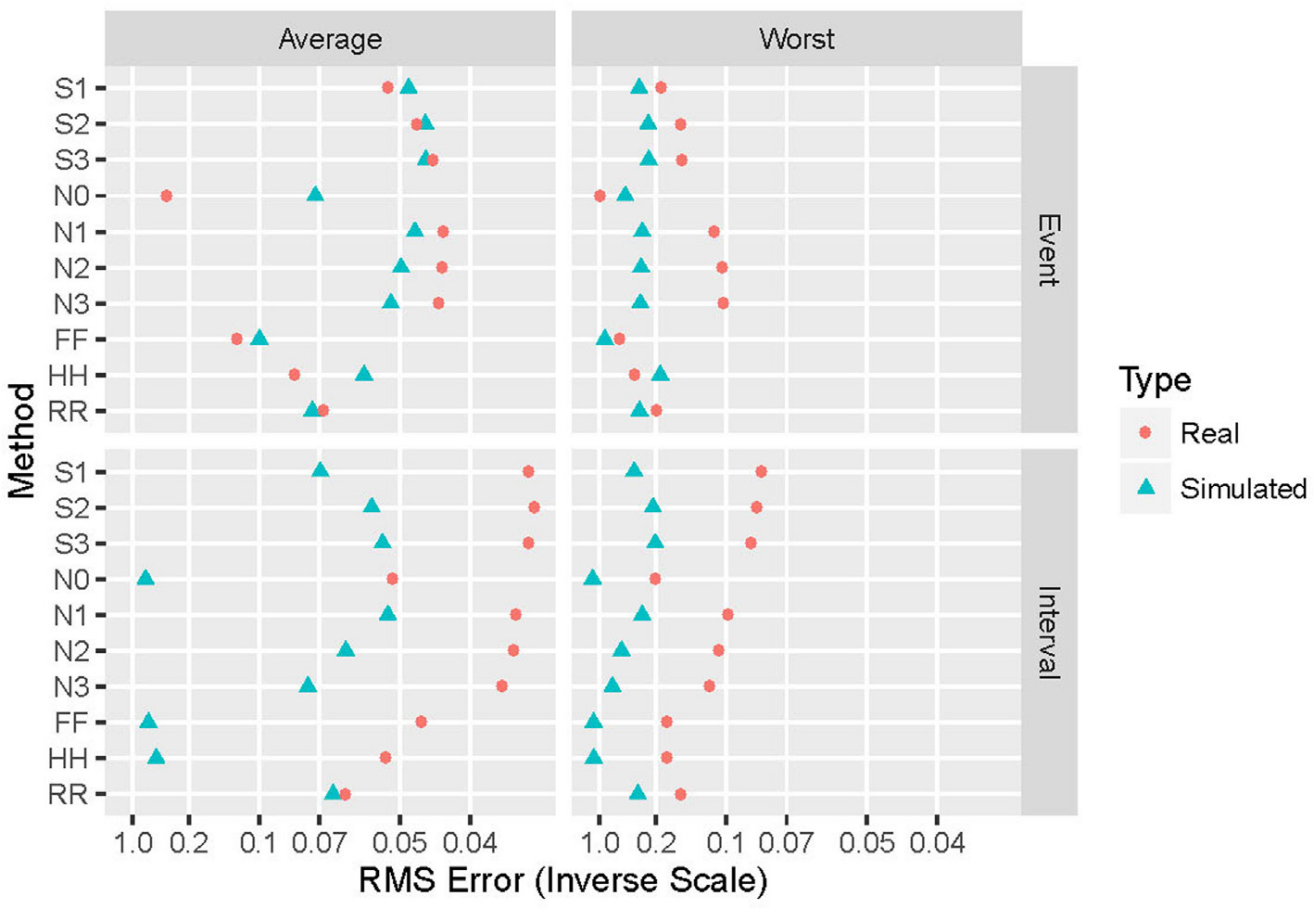
**Summary of performance of the 10 repair methods**. The 10 repair methods lie along the vertical axis in two duplicated sets: event-based above and interval-based below. Results for simulated data are in blue triangles, while those for real data re in red circles. Average performance over all distributions and power-law exponents (simulated), data sets (real), and proportion of values removed are shown on the left, while worst-case results for these same parameters appear on the right. The horizontal axis displays the reciprocal of the root mean square error (hyperbolic coordinates). This aids in distinguishing among small near-optimal values, and also places them to the right.

**Figure 5:**
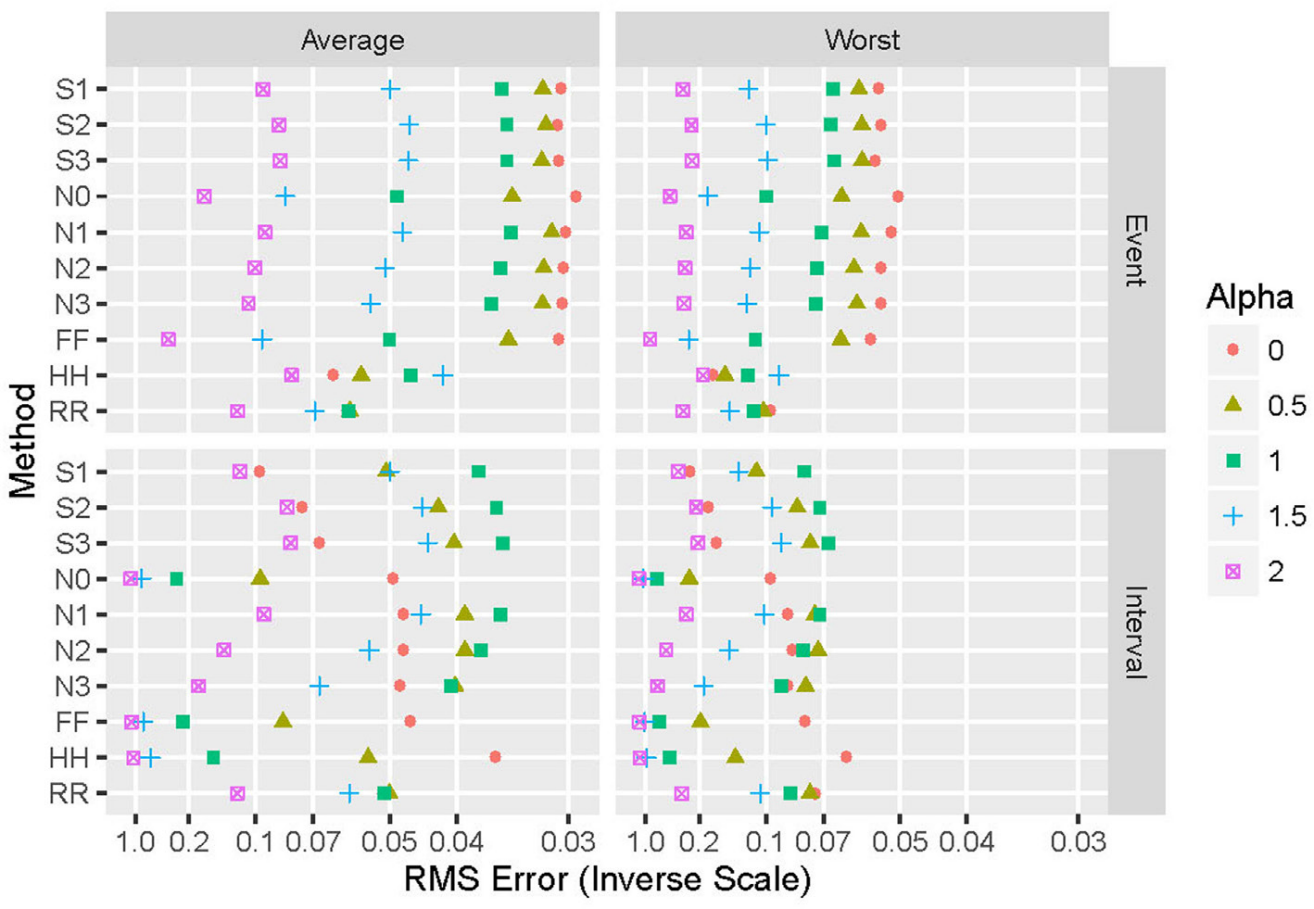
**Summary of performance of the 10 repair methods for simulated data, parameterized by design values of the power-law exponent α**. The 10 repair methods lie along the vertical axis in two duplicated sets: event-based above and interval-based below. Values of alpha include 0 (red circles), 1/2 (olive triangles), 1 (green squares), 3/2 (blue crosses), and 2 (purple boxes with X). Average performance over all distributions and proportion of values removed are shown on the left, while worst-case results for these same parameters appear on the right. The horizontal axis displays the reciprocal of the root mean square error (hyperbolic coordinates).

Generalizing from the specific case presented above (α = 2 and an exponential distribution), Figure 4 shows the performance of the various repair methods summarized over all data sets analyzed. Some methods (such as N0) are uniformly poor, others (such as S3) are good to optimum, and others (such as HH) vary widely even among these four grand averages. Whether deletion and repair are based on events or intervals changes the absolute and relative performance of the repair methods dramatically. In general, results for real intervals are much better than those for simulated ones, and for event data the real and simulated sets also yield disparate results. Figure 5 distinguishes among the power-law exponents, providing a means to evaluate repair methods when some knowledge of the power-law exponent is available.

The simulation and replacement parameters influence the performance in different ways. Results do not vary greatly with the probability distribution selected for the simulated data, suggesting that averaging over this parameter provides a useful summary of results. One exception appears to be α = 2, the largest exponent studied, for which the Gaussian and in particular the uniform distributions exhibited smaller errors for both event- and interval-based data. As expected, errors increase as *p*, the proportion of events removed and replaced, increases. Performance depends strongly on the initial (“known”) exponent. For example, for simulated, event-based data (“SE”), the worst-case performance of the HH method is the worst of all 10 methods for α < 1 and the best for α > 1; the N0 method yields nearly opposite performance.

### Event-Based Data

Replacing missing events with the midpoint of the surrounding intervals (method HH) essentially averages the two intervals. For *p* = 1% and any α, or for α ≥ 1 and any *p*, it is the best or nearly the best method. For *p* = 20% and α ≤ 1, it is the worst method, with intermediate results for this range of α and 1% < *p* < 20%. That this method yields some of the best and worst performance of all the methods arises from a number of factors. The averaging inherent in this method removes energy at high frequencies, resulting in an increase in α_2_, the measured exponent. Examining the averaged periodograms and the histograms of the exponent error shows just this effect for α ≤ 1 and *p* = 20%. For α ≥ 1, the event time course is more slowly varying by construction, and so little high-frequency energy is present in the first place. Why this method yields the best performance at α ≤ 1 and *p* = 1% is not clear. This method has the least worst-case error of all the methods.

Methods that incorporate information about the neighborhood surrounding missing events (N1–3 and S1–3) yield the best overall results, and are most useful when *a priori* information about α is not available. Results do not vary greatly among these techniques: the associated RMSE averaged over α, *p*, and distribution lie within a range of ±4%. However, method S1 has a poor worst-case error, being ranked eighth out of the 10, recommending against its use. Furthermore, the methods that do not allow scaling between template and target event patterns (N1–N3) lead to significantly increased energy at high frequencies compared with methods that do allow scaling (S1–S3). This appears in histograms of the exponent error, which show a significant negative bias, especially for large values of α. Incorporating a progressively larger neighborhood around the event improves the matches from which information is used to restore the missing event and, therefore, should improve performance. However, the larger the neighborhood, the more likely a missing event will render any particular neighborhood unusable, thus reducing the number of candidate neighborhoods. The two effects apparently cancel each other to some extent.

Basing replacement on the pair of adjacent intact intervals with the closest total duration (method N0) yields very poor performance (except for α = 0), producing the second-worst RMSE averaged over α, *p*, and distribution. Histograms of exponent error show a pronounced negative bias, especially for large values of α. This suggests that merely knowing the duration of the two intervals surrounding a missing event is not sufficient information for replacing the event.

Replacing the event according to the relative position of an event in a randomly selected pair of adjacent intact intervals (method FF) yields extremely poor performance in all cases, usually the worst of all measures, and has the worst RMSE averaged over α, *p*, and distribution. Histograms of the exponent error show a strong negative bias for larger values of a. This method completely ignores the properties of the local neighborhood of the missing event.

Simply eliminating the missing events entirely rather than attempting to replace them (method RR) yields poor results. This likely arises from a mismatch in the spliced time series following removal of the missing events, leading to spuriously increased energy at high frequencies. Histograms of the exponent error show much lower peaks than for other methods, at all values of α, corresponding to significantly increased variance for this method.

### Interval-Based Data

Replacing missing intervals with the average of the remaining intervals (method HH) introduces random shifts in the remainder of the data record, as well as changing the interval at hand. Despite this global change, it is the best or nearly the best for α = 0, and for α = 0.5 and small *p*; however, for α = 2 or large *p*, it yields the worst performance. Replacing an interval with the average over the entire record ignores the local structure, and introduces high-frequency noise. The averaged periodograms and histograms thus show a corresponding significant negative bias, which is particularly strong for large values of α.

Methods that incorporate information about the neighborhood surrounding missing events (N1–3 and S1–3) again yield the best overall results, and are most useful when *a priori* information about α is not available. For α ≥ 1, the methods that allow scaling between template and target interval patterns (S1–S3) yield better results than methods that do not (N1–N3). For α < 1 the situation is reversed. Increasing the size of the neighborhood over which matching occurs improves results for methods with scaling (S3 superior to S1), while the opposite is true for non-scaling methods (N3 inferior to N1). As with event-based data, incorporating a progressively larger neighborhood around the interval improves the matches from which information is used to restore the missing interval, but also reduces the pool of potential matches. With scaling, the former effect apparently dominates, while without scaling the latter does. The periodograms and histograms show that errors in exponent estimation are due to increased power at higher frequencies, yielding a negative bias.

With no neighborhood and no scaling (method N0), there is no structure for selecting a replacement interval, and so the method defaults to using the first valid non-deleted interval in all cases. This is similar to method HH as applied to intervals, which uses the average interval. Method N0 yields significantly poorer performance, especially as α increases.

Replacing missing intervals with randomly selected intact intervals (method FF) yields poor performance in all cases, second only to the N0 method. Like the HH and N0 methods, this approach completely ignores the properties of the local neighborhood of the missing event. Selecting random intervals introduces more variability than does employing the average (method HH). This variability is independent of the local structure and so degrades estimation of the power-law exponent.

Simply eliminating the missing events entirely rather than attempting to replace them (method RR) also yields poor results. As with event-based data, this likely arises from a mismatch in the spliced time series following removal of the missing events, leading to spuriously increased energy at high frequencies. Histograms of the exponent error again show much lower peaks than for other methods, at all values of α, corresponding to significantly increased variance for this method.

## Discussion

### Summary

Here, we investigate a number of methods for the replacement of missing values in data sets from which it is desired to extract fractal exponents *via* the slope of the power spectrum. The performance of 10 different replacement methods has been examined when applied to time series with a wide range of known exponents; real and simulated data were studied, consisting of both interval-based and event-based data. Our findings show that the most appropriate replacement method [in terms of minimizing the root mean square (RMS) error between the computed and correct exponents] depends on a variety of factors. If an estimate of the exponent is not available *a priori*, then best average performance is achieved by the S3 and N1 methods for simulated events and intervals, respectively; for best worst-case performance, methods HH and S3 are optimal. If some information on α is available, then Figure 5 provides guidance.

For both event- and interval-based data, wide differences exist between results with real and simulated data, as shown in Figure 4. Some of this discrepancy is due to the ranges of power-law exponents evaluated. The simulated data are evenly spaced between α = 0 and α = 2, while the real data have power-law exponents that lie below unity for 10 of the 12 data sets examined, and most are in the range 0.75–0.95. This clustering naturally leads to a bias toward estimated exponents in that range and, hence, different results than with the more evenly spaced simulated data. Another difference is that the real data consist of only one example, upon which 1,000 independent deletion and repair operations are performed, while for simulated data each example is different before deletion and repair. The latter thus samples much more widely from the space of possible patterns of events or intervals than does the former. This is likely to lead to poorer worst-case performance. Furthermore, simulated data span 25 different exponent-distribution combinations, leading to greater generalizability at the potential expense of less-specific connection to the data at hand.

A contingent approach potentially yields superior performance. Given a time series with events to be replaced, one can estimate the parameters of the time series that would have existed had events not been deleted. The proportion of missing events, *p*, is known. The remaining (intact) intervals can yield an estimate of the distribution of the original data; the skewness and kurtosis are easy to calculate and quantify distribution shape. Finally, a test evaluation of the fractal exponent, perhaps using method S3 that appears to be optimal overall or nearly so, provides an indication of the exponent that would have been calculated from the original data. Knowledge of *p* and estimates of α and the distribution then can locate the time series at hand in the broader parameter space and determine the optimal method for that data set. This contingent approach would also take into account potential errors induced by interpolation of *p*, the test evaluation of the fractal exponent, and estimation and interpolation of the distribution. The best method would not necessarily be the one with the least RMS error at the estimated location in parameter space, especially if performance of that method degraded significantly nearby; rather, it might be the method with the best worst-case performance in that parameter neighborhood. Finally, if characterization of the data sets at hand leads to a model not included in this paper, then simulations can be run to determine the best method to repair deleted values. This is likely to be better than using one or a few intact real data sets, as the values for each simulation are different before deletion and repair and thus span a wider portion of the data space.

### Limitations and Future Directions

We present two methods for augmenting the results discussed here. First, other methods could be employed to estimate the fractal exponent, including fitting power-law forms to wavelet transform variance using a variety of wavelets, detrended fluctuation analysis, R/S, and others. Second, the algorithms could be expanded to encompass the deletion and repair of more than one event in a row.

Physiological outliers we deal with here might occur in temporal clusters (“burstiness”). In fact, this would not be unexpected if the outliers were due to a process with fractal properties; an example would be blinking (Oh et al., 2012) as an artifact in eye-movement recordings. Our methods assume a uniform distribution for these outliers. For small rates of occurrence, this should not be a great concern, but modifications to our algorithms would be advisable if the artifact process itself is suspected to be fractal.

Our analyses have treated all data sets as monofractal: having a single fractal exponent that describes the scaling properties of the data over the entire temporal range. However, multifractal analyses can be applied to time-series data, and in fact many data sets exhibit strong evidence of multifractal behavior. Such data have different scaling exponents over different regimes of frequency and time. Thus a critical caveat is that, although our methods can be used directly for multifractal and non-stationary data, they have not been validated on such data. The methodologies that we present here can be legitimately applied only to monofractal self-affine functions. Time series thus analyzed should be examined to verify the monofractal assumption, since the effects on multifractal functions have not been investigated. Although an approximation, multifractal data can still be characterized (imperfectly) with a single scaling exponent. Thus, our data-replacement methods can be carried out under the monofractal approximation, which does not preclude later characterization as a multifractal. However, this should be considered as no more than a very crude characterization, which may lead to problems with interpretation. As an example, it could be difficult to determine to which portion of the multifractal spectrum a missing value belongs, and thus, there is a risk of mischaracterizing the replacement. This is a potentially significant drawback for multifractal applications and great care is advised in attempting such an application. Alternatively, if it is known beforehand that a given data set is multifractal (a determination that might be problematic if contaminated with anomalous values or if the data set is short), our methods might be modified to address scaling separately in each region. This was a complication that we wished to avoid in this, our initial work on the matter.

## Author Contributions

Both authors developed the background and identified the need for this study. SL performed the programming and data analysis. MS provided some data sets, helped in data interpretation, and suggested some of the algorithms that were tested.

## Conflict of Interest Statement

The authors declare that the research was conducted in the absence of any commercial or financial relationships that could be construed as a potential conflict of interest.
